# CircRNAs Are Here to Stay: A Perspective on the *MLL* Recombinome

**DOI:** 10.3389/fgene.2019.00088

**Published:** 2019-02-13

**Authors:** Anna Dal Molin, Silvia Bresolin, Enrico Gaffo, Caterina Tretti, Elena Boldrin, Lueder H. Meyer, Paola Guglielmelli, Alessandro M. Vannucchi, Geertruij te Kronnie, Stefania Bortoluzzi

**Affiliations:** ^1^Department of Molecular Medicine, University of Padua, Padua, Italy; ^2^Department of Women’s and Children’s Health, University of Padua, Padua, Italy; ^3^Department of Pediatrics and Adolescent Medicine, Ulm University Medical Center, Ulm, Germany; ^4^CRIMM, Center for Research and Innovation of Myeloproliferative Neoplasms, AOU Careggi, Department of Experimental and Clinical Medicine, University of Florence, Florence, Italy

**Keywords:** circRNA, leukemia, *MLL* rearrangements, fusion-circRNA, translocation breakpoint region, blood cells

## Abstract

Chromosomal translocations harbored by cancer genomes are important oncogenic drivers. In *MLL* rearranged acute leukemia (MLLre) *MLL/KMT2A* fuses with over 90 partner genes. Mechanistic studies provided clues of MLL fusion protein leukemogenic potential, but models failed to fully recapitulate the disease. Recently, expression of oncogenic fusion circular RNAs (f-circ) by *MLL-AF9* fusion was proven. This discovery, together with emerging data on the importance and diversity of circRNAs formed the incentive to study the circRNAs of the *MLL* recombinome. Through interactions with other RNAs, such as microRNAs, and with proteins, circRNAs regulate cellular processes also related to cancer development. CircRNAs can translate into functional peptides too. *MLL* and most of the 90 *MLL* translocation partners do express circRNAs and exploration of our RNA-seq dataset of sorted blood cell populations provided new data on alternative circular isoform generation and expression variability of circRNAs of the *MLL* recombinome. Further, we provided evidence that rearrangements of *MLL* and three of the main translocation partner genes can impact circRNA expression, supported also by preliminary observations in leukemic cells. The emerging picture underpins the view that circRNAs are worthwhile to be considered when studying MLLre leukemias and provides a new perspective on the impact of chromosomal translocations in cancer cells at large.

## The Acute Leukemia *Mll* Recombinome

Cancer genomes often harbor chromosomal translocations and specific rearrangements are recurrent in specific cancers, most often involving two specific genes or one promiscuous gene translocated with an array of different genes. In acute leukemia, the *MLL* gene (official gene symbol *KMT2A*) on chromosome 11q23 breaks and fuses with more than 90 translocation partner genes (TPGs) (Ottersbach et al., 2018), with only a few of them recurrently found in *MLL* rearrangements (MLLre).

Genomic studies revealed that *MLL* fusions are clonal and are considered early initiating leukemogenic events (Yip and So, 2013; Sanjuan-Pla et al., 2015), and very few additional mutations are needed to generate infant MLLre (Dobbins et al., 2013). Mechanistic studies of particular MLLre showed that fusion proteins are crucial for transforming potential (Reimer et al., 2017). In addition, recent data indicated that expression of the wild type *KMT2A* is dispensable for MLLre leukemic cells, whereas deletion of *KMT2D* alone or in combination with loss of *KMT2A* reduces proliferation and induces apoptosis of *MLL-AF9* transformed cells (Chen et al., 2017).

The 2017 survey of the *MLL* recombinome consortium (Meyer et al., 2018), based on DNA sequence analysis of 2,345 MLLre acute leukemia cases and literature scrutiny, identified 135 different MLLre involving 94 TPGs, with only a few genes accounting for most cases. Association of specific partner genes and/or the breakpoint position with age classes and/or with leukemia subtypes has been described (Meyer et al., 2018). *AF4* is almost exclusively (99%) found in BCP-ALL, whereas *AF9* is slightly more frequent (60%) in myeloid leukemias. Specific *MLL* TPGs are detected exclusively in leukemias of the lymphoid (e.g., *LAF4/AFF3*) or of the myeloid lineages (e.g., *SEPT6*).

*MLL* breakpoints occur preferentially in three clusters (ex-in 9, ex-in 10, ex-in 11/12) (van Dongen et al., 1999; de Jonge et al., 2005). *MLL* encodes a Lysine Methyltransferase involved in tissue-specific epigenetic activation of developmental genes. Most TPGs encode proteins of complexes that affect transcriptional elongation. Even if the functional effect has not been investigated for all *MLL* fusions, MLLre leukemias display a deeply deregulated epigenetic and transcriptional state, and the contribution of *MLL* fusions to leukemia initiation and evolution, therapy resistance and relapse is still under active investigation. An array of cellular and animal models generated to study the leukemogenic mechanisms of *MLL* fusions furthered our understanding of MLLre, but failed to fully recapitulate the human disease features (Ottersbach et al., 2018). The current state-of-the-art is controversial regarding the cell-of-origin, the timing and level of MLL fusion protein expression. Even less is known of the transcripts expressed by *MLL* fusions. Recent reports of oncogenic fusion circular RNAs (f-circ) and data emerging on circular RNAs (circRNAs, transcripts in which the splice donor site is covalently bound to an upstream acceptor site by backsplicing) in general made the drawing more complex, but also very intriguing, and opened a series of new perspectives.

Rearranged cancer genomes (promyelocytic leukemia with *PML-RAR*α, acute myeloid leukemia with *KMT2A-MLLT3* and a model of the *NPM1-ALK* fusion) (Guarnerio et al., 2016; Babin et al., 2018) express f-circ. F-circ include two sequences not present in the normal genome: the fusion junction, in which two genomic regions far apart in normal genomes are juxtaposed, and the backsplice junction, connecting in reverse order two sequences of the fusion gene. A few studies showed that f-circ can be oncogenic but only started to disclose involved mechanisms. Concurrent expression of f-circM9 and MLL-AF9 protein contributed to leukemia progression in *in vivo* and *ex vivo* models, and f-circM9 expression increased drug resistance of leukemic cells (Guarnerio et al., 2016). F-circ generated from an *EML4-ALK* fusion promoted non-small cell lung cancer development (Tan et al., 2018). The oncogenic potential of f-circ was linked to mechanisms involving the fusion protein by the observation that proliferation and TKI resistance of *BCR-ABL1* leukemic cells are enhanced by a f-circ that increases the fusion protein level. Nevertheless, none of these studies investigated the impact of rearrangements on circRNAs expressed by *MLL* and TPGs during normal hematopoiesis. In leukemic cells carrying translocations beyond generation of f-circ, also ablation or deregulation of circRNAs will occur with potential contribution to the disease.

Circularized transcripts of specific genes were reported since the 80’s, but circRNAs were re-discovered (Salzman et al., 2012; Memczak et al., 2013, 2015; Bonizzato et al., 2016) when projects coupling RNA-seq with bioinformatics methods for reads mapping appropriate to detect backsplicing showed that thousands of circRNAs are expressed by many genes with cell type-specific expression regulation (Maass et al., 2017), and differentiation stage-specificity (Rybak-Wolf et al., 2015). As reviewed recently (Memczak et al., 2013, 2015; Bonizzato et al., 2016) circRNAs are abundantly expressed in the hematopoietic compartment. CircRNAs can play important and diverse functions, including some typical of non-coding RNAs. The first function assigned to circRNAs was sponging miRNAs (Salzman et al., 2012; Memczak et al., 2013, 2015; Bonizzato et al., 2016) and indirectly regulating miRNA-target expression. In this way, some circRNAs control key miRNA-involving axes in normal developmental processes and oncogenesis (Hansen et al., 2013; Li F. et al., 2015). Other circRNAs regulate cellular processes by interacting with RNA-binding proteins (Schneider et al., 2016), scaffolding molecular complexes, as shown for circFOXO3 that controls cell cycle progression by binding p21 and CDK1 (Du et al., 2016). As long as non-coding RNAs, also circRNAs have some coding potential, and can be translated into functional peptides according to recent reports (Legnini et al., 2017; Pamudurti et al., 2017; Yang et al., 2017).

Taken together these discoveries of circRNA pervasiveness and functions prompted us to study the circRNAs expressed by genes of the *MLL* recombinome (MLL-rec) in normal blood cells. In this perspective we examined how rearrangements can result in alteration of sequences and expression level of circRNAs normally generated by *MLL* and TPGs, and provided data of MLLre leukemia in support.

## CircRnas Expressed in Normal Hematopoiesis by Genes of the *Mll* Recombinome

The MLL-rec analyzed here included *MLL* plus 94 TPGs (Supplementary Table 1): 75 genes of rearrangements disclosed by Meyer et al. (2018) and 19 genes previously reported in the literature collected in the same study. Almost 93% of 2,345 leukemia cases reported in Meyer et al. (2018) resulted from *MLL* fusions with one of 12 highly recurrent TPGs (*AF4/AFF1, AF9/MLLT3, ENL/MLLT1, AF10/MLLT10, BCS1L/PTD, ELL, AFDN/AF6/MLLT4, EPS15, AF1Q/MLLT11, SEPT6, AF17/MLLT6*, and *SEPT9*), that will be cited from now on using the official gene name.

To obtain a data-driven picture of the circular transcriptome of MLL-rec genes in the hematopoietic compartment, we analyzed our RNA-seq dataset regarding normal hematopoiesis (available at GEO series ID: GSE110159 and upon request): 15 samples from healthy donors, including 3 of CD34+ cells purified from cord blood, and 12 of B-, T-cells and monocytes FACS sorted from PBMCs (4 different donors per cell type). RNA-seq data were obtained from ribo-depleted RNA with Illumina^®^ HiSeq2000 (average depth of 145 M paired end 100 nt reads per sample, 66% of reads passed quality control). CircRNAs were detected and quantified using CirComPara v0.3 (Gaffo et al., 2017), implementing 6 circRNA detection methods (CIRI, Findcirc, CIRCexplorer2+Star, CIRCexplorer2+Segemehl, CIRCexplorer2+BWA, CIRCexplorer2+TopHat). CirComPara identified 41,515 circRNAs expressed from 8,138 individual genes. We focused on the subset of 16,606 circRNAs with high expression in at least one cell type (maximum of the averages per cell type in the top 40% highest values), which derived from 5,170 genes.

For 25 of the 95 genes in the MLL-rec, no circRNAs were detected in our dataset and 16 had only circRNAs expressed at low level. Interestingly, 54 genes of the recombinome expressed at least one abundant circRNA, for a total of 327 circRNAs (Supplementary Table 2), which were further investigated.

The expression level of these circRNAs was not different from the 16,005 circRNAs expressed by the 5,116 non-MLL-rec genes of the human genome (Supplementary Figure 1). MLL-rec genes expressed several circRNAs at very high levels: the 33 most expressed circRNAs (Supplementary Figure 2) derived from 21 genes, including *AFF1* and *EPS15*, which presented circRNAs overexpressed in stem cells; and *SEPT6* and *SEPT9*, whose circRNAs were detected only in mature cells, with marked overexpression of circSEPT9 17:77402059–77402703:+ in monocytes. CircAFF3 2:100006632–100008932:- and circAFF4 5:132892164–132893118:-, both from *MLL* TPGs only found in leukemias of the lymphoid lineage, were upregulated in B-cells. Four genes contributed several circRNAs to the group of the most highly expressed: 10 circPICALM were mostly upregulated in monocytes, albeit circPICALM 11:86007542–86026367:- and 11:86022367–86031611:- were more abundant in stem cells; 2 circME2 were upregulated in stem cells; 2 circARHGAP26 were upregulated in monocytes; 2 circPDS5A were upregulated in B-cells.

**FIGURE 1:**
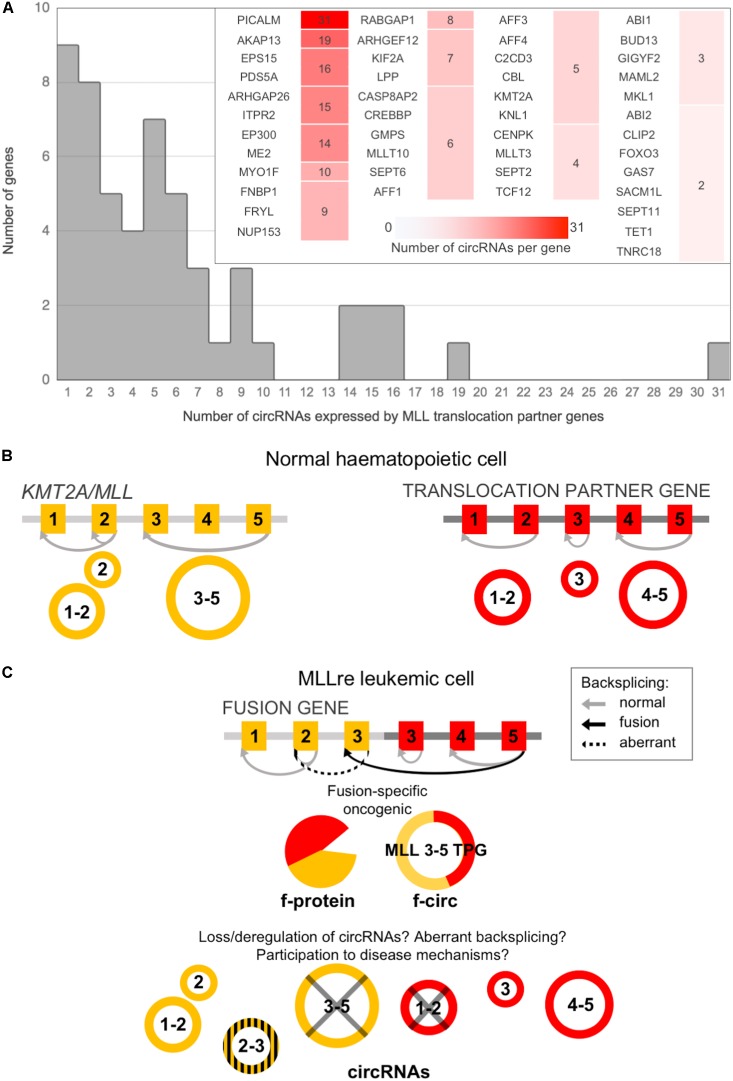
CircRNA expression by *MLL/KMT2A* and by *MLL* translocation partner genes (TPGs) in normal hematopoiesis can be altered in leukemic cells. **(A)** Distribution of the number of circRNAs per gene expressed in normal blood cells by the *MLL* recombinome (the detail shows genes with multiple isoforms); **(B)** *MLL/KMT2A* and a TPG can produce, in normal blood cells, several circRNAs; **(C)** CircRNA expression can be affected by the fusion: beyond the generation of fusion-specific products (protein and circRNAs), the absence in the fusion gene of the exons undergoing circularization and/or of the intronic sequences flanking the backsplicing with regulatory functions can deregulate or abolish circRNA expression; in addition, aberrant circRNAs can be generated by activation of “cryptic” backsplicing sites.

MLL-rec genes compared to non-MLL-rec genes had a significantly larger number of circular isoforms per gene (6.06 vs. 3.13 circRNAs per gene in average; *t*-test *p*-value 2.317 E–04), with a distribution of the number of circRNAs per gene significantly shifted toward higher values (Chi-squared test *p*-value 1.791 E–06; Supplementary Figures 3A,B). Indeed, 9 of the recombinome genes expressed only one circRNA each, and 45 (83%) had multiple circular isoforms in blood cells (Figure 1A). The genes with the highest numbers of isoforms were *PICALM* (31 circRNAs), *AKAP13* (19), *EPS15* (16), *PDS5A* (16), *ARHGAP26* (15), *ITPR2* (15), *EP300* (14), *ME2* (14), and *MYO1F* (10). Moreover, *MLLT10*, *SEPT6* and *AFF1* had 6 circRNAs each, and *MLL3* expressed 4 circRNAs.

Similar numbers of circRNAs were expressed in the different mature populations by MLL-rec (205, 244, and 210 in B-, T-cells and in monocytes, respectively), and only 94 circRNAs in the stem population (Supplementary Figure 4A). Apart from 44 circRNAs expressed in all cell types, 16 from 10 genes (*CENPK*, *LPP*, *ITPR2*, *TCF12*, *CLTC*, *FNBP1*, *FRYL*, *SEPT11*, *SEPT6*, and *MLLT10*) were detected only in stem cells (circCENPK 5:65528452–65529145:- was the most abundant), and 233 had expression restricted to one or more mature populations, being mostly detected in all the mature populations (80) or in lymphocytes (40).

Most genes displayed cell type-specific alternative circularization patterns (Supplementary Figure 4B). Of 16 stem cell-specific circRNAs, circCLTC 17:59668941–59669222:+ was expressed by a gene with circRNAs only in stem cells, and 15 derived from genes with other circular isoforms detected in mature cell types. The three most abundant of the 16 different circEPS15 had different expression profiles (Supplementary Figure 4C): circEPS15 1:51402435–51408332:- was expressed in all cell types with a slight upregulation in stem cells, whereas circEPS15 1:51394381–51448135:- was upregulated only in monocytes, and circEPS15 1:51405905–51408332:- was more abundant in B- and T-cells than in monocytes, and not detected in stem cells.

A scrutiny of the two main circRNA-indexing databases, CircBase and circRNADB, showed that 2,433 circRNAs were previously reported for 85 of 95 genes in the MLL-rec, including all the 54 with highly expressed circRNAs, and all but *ACER1* and *PFDN4* of the 70 genes with circRNAs detected in our data. Moreover, for 17 (out of 25) genes in the MLL-rec for which we did not observe appreciable circRNA expression in blood cells, the DBs reported circRNAs mostly in other tissues, such as brain (e.g., *ARHGEF17*) or muscle (e.g., *FLNC*). Even if a direct comparison of our data with data reported in the DBs is not feasible, findings concordantly indicate that circRNAs are expressed from the overwhelming majority of genes in the MLL-rec, with complex patterns of alternative circularization in many cases.

Of note, 63 new circRNAs were detected at high expression in blood populations investigated in this study, which are currently not annotated in the DBs (Supplementary Table 2). Newly detected circRNAs derived from 26 genes of the MLL-rec, including *MLLT10*, *MLLT3*, *EPS15* and *SEPT6*, and comprised 15 circPICALM and circRNAs with expression restricted to stem cells or to a specific mature population.

## Translocations Can Impact CircRNA Expression From *Mll/Kmt2A* and Tpgs

As described above, *KMT2A* and most of TPGs observed in acute leukemias do express circRNAs in normal blood cells (Figure 1A). In MLLre leukemias the rearrangement of *KMT2A* with a TPG leads to the formation of a “fusion gene.” When *KMT2A* is expressed, also the downstream partner gene may be expressed resulting in a transcript that potentially is translated in a fusion protein (Ayton and Cleary, 2001; So et al., 2003). Alongside also f-circ may be produced from chimeric fusion genes (Guarnerio et al., 2016), as reviewed in Bonizzato et al. (2016). In addition, the breaking and fusion of *KMT2A* and TPGs may alter the formation of circRNAs from exons proximal to the breakpoint of the translocated genes. Information on the *KMT2A* and TPGs specific exons that undergo circularization in normal hematopoiesis (Figure 1B) is critical to understand how a specific rearrangement can impact circRNA expression (Figure 1C). The breakpoint position defines which genomic regions are retained in the fusion gene, determining not only if and how f-circ are generated. The breakpoint position can also affect the circRNAs “normally” generated by the partner genes influencing their expression level or possibly activating cryptic backsplicing sites.

For *KMT2A* and of three of the main TPGs we further examined in detail circRNAs expression in normal blood cells in relation to potential breakpoint regions. In addition, we provided preliminary data of MLLre leukemia using RNA-seq data of the THP1 cell line with *KMT2A-MLLT3* (*MLL-AF9)* fusion (Guarnerio et al., 2016) and unpublished data of two specimens of infant ALL with *KMT2A-MLLT1* (*MLL-ENL)* fusions (Supplementary Table 3).

**FIGURE 2:**
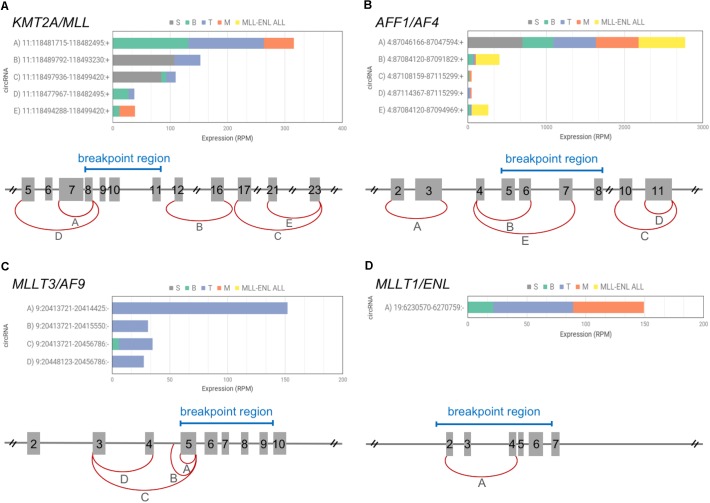
CircRNAs expressed by *KMT2A* and 3 TPGs among the most recurrent in acute leukemias. The picture includes a schematic diagram of exon/intron structure of *KMT2A* gene **(A)** and of the 3 selected TPGs: *AFF1* **(B)**, *MLLT3* **(C)**, *MLLT1* **(D)**, that give rise to fusion genes in acute leukemias; exon numbering and relevant breakpoint regions (blue bars) are indicated for each gene (reference transcripts: ENST00000534358.6, ENST00000307808.10, ENST00000380338.8, and ENST00000252674.8); the most expressed circRNAs are shown by red arches, indicating the position of the backsplice ends. For each gene, different letters identify circRNAs for which the barplot shows average expression in the four normal blood cell populations (S, stem cells; B, B-cells; T, T-cells; M, monocytes), as well as in leukemic cells bearing *KMT2A-MLLT1* fusion (*MLL-ENL* ALL).

In normal hematopoiesis, *KMT2A* produced 5 different circRNAs (Figure 2A). The most abundant circKMT2A (11:118481715–118482495:+; exons 7–8) was expressed in all mature populations, with upregulation in both B- and T-cells, while it was absent in the stem population, in which two other circKMT2A isoforms were detected. The formation of two wild type circKMT2A isoforms (exons 5–8 and 7–8) are potentially perturbed in MLLre leukemias, considering that the recurrent breakpoint region includes exon 8. The other three circKMT2A expressed in normal hematopoiesis (exons 12–16, 17–23, 21–23) will probably not be formed from the fusion gene, since they originate from exons not retained in most rearrangements. Thus, all circRNAs highly expressed in normal cells by *KMT2A* may be downregulated or even absent in the leukemic cells. In line, none of the circRNAs expressed in normal hematopoiesis by *KMT2A* was detected, neither in the THP1 cell line (in which two fusion circRNAs were previously detected by RT-PCR, and albeit supported by few reads, from RNA-seq data), nor in any of the two samples with *KMT2A-MLLT1* fusion. Of note, *KMT2A* exons 5 or 7 are the starting ends backspliced both in normal hematopoiesis (joined with exon 8 in two of the five observed circKMT2A isoforms) and in THP1 cells (joined with *MLLT3* exon 6 in the two f-circM9 isoforms) (Guarnerio et al., 2016). If and how the reported oncogenic potential of f-circM9 (*KMT2A* exon 7 – *MLLT3* exon 6) is related to the function of the most expressed circKMT2A (*KMT2A* exons 7–8), that is fully contained in the f-circM9, remains to be determined.

The three TPGs most recurrent in acute leukemias (*AFF1, MLLT3, MLLT1*) expressed one to 5 circRNAs each in normal hematopoiesis (Figure 2B–D). *AFF1* presented 5 circRNAs, with circAFF1 4:87046166–87047594:+ (exons 2–3) contributing alone to 90% of circular expression of the gene. The same circAFF1 was one of the most expressed in the sample set, it presented a remarkable upregulation in stem cells compared to mature cells according to our data, and was found upregulated in common lymphoid precursors and in monocytes (Nicolet et al., 2018). The *AFF1* recurrent breakpoint region position in MLLre leukemias reveals that this circAFF1 will likely not be generated from the chimeric gene. The expression of the other 4 circAFF1 will depend on the exact position of the breakpoint (Figure 2B).

*MLLT3* had 4 circular isoforms, almost all specific of T-cells. The second most expressed circMLLT3 contains part of intron 4, as observed for other circRNAs (Li Z. et al., 2015). These circRNAs will not be generated from the fusion gene due to the position of the breakpoints, either falling within the sequence undergoing circularization or eliminating all circularized exons from the fusion transcripts (Figure 2C). None of the T-specific circMLLT3 isoforms was detected in *KMT2A-MLLT3* THP1 cells.

Also circMLLT1 9:6230570–6274759:- (exons 2–4), expressed in mature populations, will not be generated from the *KMT2A-MLLT1* derivative, since the breakpoints of *t*(11,19) leukemias either abolish the *MLLT1* exons undergoing circularization or fall in the intron flanking the backspliced exon 2. In line with our assumption, this circRNA was not detected in leukemic cells (Figure 2D) with two different *KMT2A-MLLT1* fusions (*KMT2A* exon 9 – *MLLT1* exon 6, and *KMT2A* exon 8 – *MLLT1* exon 4 fusions, Supplementary Table 3).

Here, we analyzed one of the main *KMT2A*-TPG fusions, and the translocations indeed impacted circRNAs expressed from *KMT2A* and its TPG. Highly informative data on leukemias with *KMT2A-MLLT1* fusions were fully in agreement with observations (based on a lower depth) in THP1 cells. Little is known about the other TPG-*KMT2A* derivatives.

To generalize, based on circRNA and breakpoint respective position, four scenarios can occur: (1) The exons undergoing circularization are retained in the *KMT2A*-TPG derivative (e.g., exons in the 5′ region of *KMT2A* or in the 3′ region of the TPG retained in the fusion gene), circRNAs can be still generated; (2) The exons undergoing circularization are not retained, the circRNAs can be abolished; (3) The breakpoint is located between the backspliced ends, the absence in the fusion gene of one or more exons normally undergoing circularization will prevent the formation of circRNAs, possibly favoring the generation of f-circ; (4) The breakpoint is located in an intron flanking one of the backsplice ends abolishing specific *in cis* sequence elements (Alu and inverted repeats) that favor the backsplicing (Jeck et al., 2013; Bonizzato et al., 2016), and/or sequences recognized by *in trans* regulatory factors, such as QKI and ADAR1, which promotes (Conn et al., 2015) and suppresses (Rybak-Wolf et al., 2015) circularization, respectively. We are well aware that our model of how translocations can impact expression of circRNAs from fused genes is hampered by simplicity. Position-effects, as well as deep genome-wide epigenetic and transcriptional deregulation in leukemic cells can affect circRNAs expressed from the fused genes as well as circRNAs from other loci.

## Conclusion and Future Directions

Data emerging from recent literature and from the present study collectively show that *KMT2A* and TPGs express many circRNAs, possibly playing important functions and being perturbed in cells bearing rearrangements. According to our observations, depending on the position of the breakpoint respective to the backspliced sequences, translocations can impact expression of circRNAs in addition to generating f-circ. Our data on circRNAs expressed from genes of the *MLL* recombinome are instrumental to analyze alterations of circRNAs from rearranged genes of MLLre leukemias and f-circ generation. It is known that f-circ can be oncogenic, but the involved mechanisms remain essentially unknown. F-circ and the other circRNAs expressed from *KMT2A* and TPGs might share part of their sequences and play similar functions (e.g., through common protein or nucleic acids interactors, or due to overlapping coding potential). F-circ were previously shown to reinforce the oncogenic potential of fusion proteins, perhaps cooperating to the same mechanisms (Babin et al., 2018). A parallel line of evidence showed that loss of both *KMT2A* and *KMT2D* impair survival pathways in leukemic cells (Chen et al., 2017), implicating them in oncogenesis. In this view, beyond f-circ, also circRNAs generated from the non-translocated alleles are critical candidates to be investigated for participation to disease mechanisms. Further, f-circ and circRNAs in general share exons with linear transcripts, and circularization is associated with exon skipping (Kelly et al., 2015). The equilibrium perturbed by translocations can involve a series of transcripts including “normal” as well as aberrant circular and linear transcripts, all linked in an intricate network of similar sequences and functions and interdependent biogenesis.

In conclusion, next to the studies of fusion proteins as oncogenic drivers of leukemias with *MLL* rearrangements, we stress the need of a molecular characterization of circRNAs expressed by fusion genes, *KMT2A* itself and its TPGs. A first direction could be to clarify the participation of circRNAs to the molecular complexes involving *KMT2A*, and to define circRNA molecular interactions with regulators, such as microRNAs, of *KMT2A* or of its interactors involved in leukemogenesis. The perspective emerging from this pilot study on MLLre acute leukemia is presumably valid for most of the driver chromosomal fusions occurring in cancer cells.

## Ethics Statement

All experiments involving human material followed the principles outlined in the Helsinki Declaration. The study has been approved by the ethics committees of Padova University Hospital, Ulm University Medical Center and of AOU Careggi (Florence) and written informed consent was obtained from all subjects.

## Author Contributions

GtK and StB conceived the study. ADM, EG, and StB contributed bioinformatics methods and performed data analysis. SiB, CT, and StB performed comparative public data analysis. ADM, StB, and GtK wrote the manuscript. StB, ADM, and SiB made the figures. EB, LM, PG, and AV provided data and revised the manuscript. All authors approved the final manuscript.

## Conflict of Interest Statement

The authors declare that the research was conducted in the absence of any commercial or financial relationships that could be construed as a potential conflict of interest.
