# Supplementary material for: CircRNAs Are Here to Stay: A Perspective on the MLL Recombinome
Source: Front Genet. 2019 Feb 13;10:88. doi: 10.3389/fgene.2019.00088 (PMC6382020; doi:10.3389/fgene.2019.00088)
Supplement: Supplementary file 1 [file Data_Sheet_1.pdf]

CircRNAs are here to stay: A perspective on the MLL recombinome

Anna Dal Molin, Silvia Bresolin, Enrico Gaffo, Caterina Tretti, Elena Boldrin, Lueder H. Meyer, Paola Guglielmelli, Alessandro M. Vannucchi, Geertruy te Kronnie, Stefania Bortoluzzi

Supplementary material

Supplementary Table 1. Data on the 94 MLL TPGs considered in this study, using the 2017 update of the MLL recombinome as reference. The list includes 75 genes of rearrangements associated to recurrence data in 2,345 acute leukemia cases of the reference study, plus 19 genes previously recorded in literature (see Meyer et al. 2018 and references therein). For all the genes, including those cited in literature using aliases, the official gene name was used in this study.

Official gene name	Alias	Number of cases (Meyer et al. 2018)
AFF1	AF4	839
MLLT3	AF9	449
MLLT1	ENL	302
MLLT10	AF10	197
BCS1L	PTD	107
ELL		97
AFDN	AF6 MLLT4	95
EPS15		38
MLLT11	AF1Q	23
SEPT6		17
MLLT6	AF17	14
SEPT9		13
AFF3	LAF4	8
TET1	LCX	6
SEPT5		5
ABI1		4
KNL1		4
MAML2		4
MYO1F		4
PICALM		4
TNRC18		4
FLNA		3
NEBL		3
ABI2		2
ACTN4		2
AFF4	AF5	2
BTBD18		2
CBL		2
CEP170B		2
CIP2A	KIAS1524	2
CREBBP		2
DCP1A		2
FOXO3		2
SEPT11		2

ACACA		1
ACER1		1
AKAP13		1
AP2A2		1
ARHGAP26		1
ARHGEF12		1
ARHGEF17		1
BCL9L		1
BUD13		1
C2CD3		1
CASP8AP2		1
CEP164		1
CLTA		1
CLTC		1
CT45A2		1
DCPS		1
EEFSEC		1
FNBP1		1
GAS7		1
GMPS		1
KIF2A		1
LAMC3		1
LOC100131626		1
ME2		1
MKL1		1
MYH11		1
NOX4		1
NRIP3		1
NUP153		1
PDS5A		1
PFDN4		1
PRPF19		1
PRRC1		1
RABGAP1		1
RUNDC3B		1
SEPT2		1
SMAP1		1
TCF12		1
TOP3A		1
VAV1		1
GIGYF2		1
ASXL2		-
CENPK	FKSG14	-
CLIP2		-
DAB2IP	AF9Q34	-
EP300		-
FLNC		-
FOXO4	AFX	-
FRYL		-
GPHN		-
ITPR2		-
LOC100128568		-
LPP		-
MAPRE1		-
NCKIPSD	AF3P21	-
SACM1L		-
SARNP	CIP29	-
SH3GL1	EEN	-
SORBS2	ARGBP2	-
ZFYVE19	MPFYVE	-

Supplementary Table 2. Data on the 327 circRNAs with high expression derived from the *MLL* recombinome, indicating backsplice end positions, the gene of origin and the average expression per cell type (RPM); a flag "new" in the last column identifies circRNAs not annotated in CircBase and CircRNADB. CircRNAs are ordered by the highest mean expression among cell types.

CircRNA	Gene ID	Gene name	Mean expression (RPM)				Found in DBs
			CD34+	B-cell	T-cell	Monocyte	
11:86007542-86031611:-	ENSG00000073921	PICALM	334.1	296.3	691.0	1726.1	new
11:85996826-86003451:-	ENSG00000073921	PICALM	494.8	233.1	321.6	1546.1	
11:85996826-86031611:-	ENSG00000073921	PICALM	127.0	185.9	357.0	1404.9	new
11:85974708-85983973:-	ENSG00000073921	PICALM	449.5	302.3	663.9	1274.7	
11:86011030-86031611:-	ENSG00000073921	PICALM	994.5	338.6	476.7	1189.5	new
4:39913611-39925933:-	ENSG00000121892	PDS5A	700.3	949.1	452.1	648.2	
5:132892164-132893118:-	ENSG00000072364	AFF4	266.9	884.5	429.1	142.7	
11:86022367-86031611:-	ENSG00000073921	PICALM	834.4	482.2	665.0	745.3	new
16:3850297-3851009:-	ENSG00000005339	CREBBP	366.4	199.9	291.3	819.0	
4:87046166-87047594:+	ENSG00000172493	AFF1	694.7	397.1	541.3	547.5	
5:142885298-142932125:+	ENSG00000145819	ARHGAP26	90.8	0.0	205.1	576.8	
5:143037196-143057747:+	ENSG00000145819	ARHGAP26	183.0	26.8	174.4	561.7	
12:26550247-26602706:-	ENSG00000123104	ITPR2	539.4	0.0	6.1	0.0	
11:85990250-86031611:-	ENSG00000073921	PICALM	127.6	18.4	102.6	538.0	new
15:40628070-40650819:+	ENSG00000137812	KNL1	149.9	4.8	0.0	515.9	
10:68644698-68647005:+	ENSG00000138336	TET1	490.0	26.5	23.1	17.3	
11:119284969-119285566:+	ENSG00000110395	CBL	0.0	184.0	447.6	484.1	
11:86007542-86026367:-	ENSG00000073921	PICALM	483.3	20.9	3.3	54.8	new
2:100006632-100008932:-	ENSG00000144218	AFF3	68.2	466.7	0.0	45.4	
11:85974708-86031611:-	ENSG00000073921	PICALM	232.7	52.9	77.4	462.8	new
19:8536252-8540028:-	ENSG00000142347	MYO1F	0.0	37.5	208.3	413.1	
6:89846562-89857199:+	ENSG00000118412	CASP8AP2	398.8	151.5	104.4	125.8	
17:77402059-77402703:+	ENSG00000184640	SEPT9	0.0	24.0	112.5	382.1	
6:17665239-17669546:-	ENSG00000124789	NUP153	356.5	70.5	36.2	66.2	
18:50918110-50924212:+	ENSG00000082212	ME2	354.5	57.6	159.6	223.0	
11:96091892-96093517:-	ENSG00000184384	MAML2	129.3	44.4	351.8	254.1	
6:108663455-108664889:+	ENSG00000118689	FOXO3	169.2	56.6	71.5	348.4	
X:119640692-119653040:-	ENSG00000125354	SEPT6	0.0	340.6	246.1	225.6	
1:51402435-51408332:-	ENSG00000085832	EPS15	338.5	223.3	266.1	285.3	
11:86012281-86031611:-	ENSG00000073921	PICALM	0.0	71.6	29.1	317.9	new
18:50918110-50921187:+	ENSG00000082212	ME2	313.0	90.5	96.2	32.1	
4:39837856-39842056:-	ENSG00000121892	PDS5A	131.6	311.0	129.6	112.5	
2:232787150-232796221:+	ENSG00000204120	GIGYF2	254.8	143.9	254.7	303.4	
4:48684673-48710698:-	ENSG00000075539	FRYL	218.3	184.5	287.0	64.4	
22:41125864-41140257:+	ENSG00000100393	EP300	0.0	34.5	39.9	270.2	
5:143054439-143134105:+	ENSG00000145819	ARHGAP26	0.0	0.0	25.3	266.7	
9:36197551-36204179:+	ENSG00000122705	CLTA	261.9	70.7	38.0	0.0	

1:51394381-51448135:-	ENSG00000085832	EPS15	61.6	24.6	30.7	242.3	
5:143054439-143147381:+	ENSG00000145819	ARHGAP26	0.0	0.0	37.0	238.9	
5:143054439-143057747:+	ENSG00000145819	ARHGAP26	107.3	94.6	184.4	235.5	
22:40429606-40435554:-	ENSG00000196588	MKL1	0.0	217.9	12.9	44.0	
11:119273868-119274953:+	ENSG00000110395	CBL	214.6	217.2	111.1	34.5	
15:40641379-40650819:+	ENSG00000137812	KNL1	0.0	0.0	0.0	215.2	
11:85996826-86001158:-	ENSG00000073921	PICALM	212.3	29.1	39.9	98.8	
11:120474560-120477526:+	ENSG00000196914	ARHGEF12	208.2	33.7	47.8	0.0	
6:17674905-17675770:-	ENSG00000124789	NUP153	0.0	70.3	208.1	66.5	
19:8551740-8553437:-	ENSG00000142347	MYO1F	0.0	25.8	122.6	196.4	
18:50918110-50940386:+	ENSG00000082212	ME2	0.0	62.6	44.7	193.7	
7:74356410-74357477:+	ENSG00000106665	CLIP2	193.7	76.4	35.5	89.1	
5:65528452-65529145:-	ENSG00000123219	CENPK	189.6	0.0	0.0	0.0	new
18:50918110-50932360:+	ENSG00000082212	ME2	0.0	56.2	115.4	185.2	
3:155893518-155911279:+	ENSG00000163655	GMPS	129.6	32.3	59.9	183.8	
12:26550247-26655852:-	ENSG00000123104	ITPR2	178.1	26.9	43.5	57.9	
6:89862126-89871388:+	ENSG00000118412	CASP8AP2	164.4	42.8	38.5	0.0	
11:85974708-86003451:-	ENSG00000073921	PICALM	0.0	4.8	22.3	164.0	
3:188225405-188341719:+	ENSG00000145012	LPP	161.3	0.0	0.0	0.0	new
12:26483490-26483697:-	ENSG00000123104	ITPR2	161.0	0.0	0.0	0.0	new
9:122957011-123020459:+	ENSG00000011454	RABGAP1	161.0	7.2	0.0	85.3	
11:85981745-85996929:-	ENSG00000073921	PICALM	84.5	17.8	125.5	159.2	
4:39874289-39877153:-	ENSG00000121892	PDS5A	0.0	158.1	91.6	0.0	
15:57034277-57034527:+	ENSG00000140262	TCF12	156.5	0.0	0.0	0.0	new
22:41172499-41173784:+	ENSG00000100393	EP300	0.0	153.9	27.6	22.2	
11:74132844-74133557:-	ENSG00000168014	C2CD3	0.0	153.1	88.7	0.0	
9:20413721-20414425:-	ENSG00000171843	MLLT3	0.0	0.0	150.5	0.0	
5:132883340-132885119:-	ENSG00000072364	AFF4	0.0	148.5	95.5	24.7	
11:74138720-74139828:-	ENSG00000168014	C2CD3	0.0	5.7	148.3	34.5	
3:53290791-53292827:-	ENSG00000272886	DCP1A	0.0	79.2	95.3	146.8	new
4:39898389-39902460:-	ENSG00000121892	PDS5A	107.3	139.9	52.5	0.0	
12:26481131-26655852:-	ENSG00000123104	ITPR2	0.0	44.8	63.7	139.4	new
11:85981129-86031611:-	ENSG00000073921	PICALM	0.0	9.0	54.9	138.2	new
5:142885298-142913293:+	ENSG00000145819	ARHGAP26	137.2	0.0	111.4	120.9	new
9:123070350-123076762:+	ENSG00000011454	RABGAP1	135.4	13.5	44.8	0.0	
11:86000643-86003451:-	ENSG00000073921	PICALM	134.5	7.2	24.7	0.0	
11:118481715-118482495:+	ENSG00000118058	KMT2A	0.0	131.6	131.6	51.9	
10:21681332-21713950:+	ENSG00000078403	MLLT10	131.0	10.1	0.0	0.0	
11:86011030-86026367:-	ENSG00000073921	PICALM	131.0	8.9	0.0	0.0	new
3:128262128-128264781:+	ENSG00000132394	EEFSEC	0.0	129.7	62.2	19.4	
6:89853166-89857199:+	ENSG00000118412	CASP8AP2	127.6	94.1	24.9	0.0	
9:129978465-129994958:-	ENSG00000187239	FNBP1	127.6	64.6	35.5	0.0	
4:48684673-48687160:-	ENSG00000075539	FRYL	72.1	99.9	50.6	124.5	
1:51405905-51408332:-	ENSG00000085832	EPS15	0.0	122.2	107.9	36.2	
4:39841948-39844801:-	ENSG00000121892	PDS5A	0.0	116.2	63.1	0.0	
11:116757146-116758407:-	ENSG00000137656	BUD13	114.8	15.8	36.5	25.0	

3:155910692-155914570:+	ENSG00000163655	GMPS	0.0	63.8	83.6	113.1	
22:41125864-41135906:+	ENSG00000100393	EP300	0.0	0.0	20.7	113.1	
11:85981129-85982003:-	ENSG00000073921	PICALM	112.8	59.8	15.2	33.5	
11:85981129-86007583:-	ENSG00000073921	PICALM	111.3	0.0	0.0	17.3	
6:89854817-89857199:+	ENSG00000118412	CASP8AP2	111.3	52.9	37.5	17.2	
15:57231152-57234107:+	ENSG00000140262	TCF12	0.0	24.7	110.9	88.3	
6:17665239-17688618:-	ENSG00000124789	NUP153	110.2	9.0	0.0	0.0	
12:26550247-26632059:-	ENSG00000123104	ITPR2	109.9	0.0	0.0	0.0	
17:37179260-37181356:-	ENSG00000278540	ACACA	109.6	0.0	14.6	0.0	
17:59668941-59669222:+	ENSG00000141367	CLTC	109.6	0.0	0.0	0.0	new
3:188341663-188406313:+	ENSG00000145012	LPP	0.0	109.1	24.0	0.0	
12:26377741-26377995:-	ENSG00000123104	ITPR2	108.8	0.0	0.0	0.0	new
9:123084643-123090385:+	ENSG00000011454	RABGAP1	108.8	59.5	4.9	0.0	
9:130037463-130037763:-	ENSG00000187239	FNBP1	108.8	0.0	0.0	0.0	new
4:48540353-48551578:-	ENSG00000075539	FRYL	107.5	0.0	0.0	0.0	new
4:76996425-77020670:+	ENSG00000138758	SEPT11	107.4	0.0	0.0	0.0	
9:129902869-129929695:-	ENSG00000187239	FNBP1	107.4	0.0	0.0	0.0	
11:118489792-118493230:+	ENSG00000118058	KMT2A	107.3	0.0	45.1	0.0	
10:26759062-26770345:-	ENSG00000136754	ABI1	0.0	9.0	20.3	106.6	
11:85983866-86031611:-	ENSG00000073921	PICALM	0.0	0.0	69.7	106.4	new
15:85521428-85543955:+	ENSG00000170776	AKAP13	106.0	0.0	6.2	0.0	
X:119618288-119618494:-	ENSG00000125354	SEPT6	104.2	0.0	0.0	0.0	new
6:17646067-17649300:-	ENSG00000124789	NUP153	0.0	101.8	25.4	0.0	
10:26765218-26770345:-	ENSG00000136754	ABI1	0.0	86.1	31.7	101.8	
3:155910692-155925366:+	ENSG00000163655	GMPS	0.0	24.4	22.2	100.4	
4:48590659-48605833:-	ENSG00000075539	FRYL	0.0	0.0	10.4	100.4	
4:39869394-39890364:-	ENSG00000121892	PDS5A	55.0	46.1	69.3	100.0	
12:26621123-26655852:-	ENSG00000123104	ITPR2	0.0	22.5	36.4	99.1	new
15:85575131-85585823:+	ENSG00000170776	AKAP13	0.0	20.6	0.0	98.3	
22:40552106-40594735:-	ENSG00000196588	MKL1	0.0	44.2	57.7	96.2	
1:51461091-51465326:-	ENSG00000085832	EPS15	0.0	95.9	67.5	0.0	
5:143054439-143121147:+	ENSG00000145819	ARHGAP26	0.0	0.0	12.6	95.7	
11:74132844-74139828:-	ENSG00000168014	C2CD3	0.0	30.1	22.2	95.7	
10:21673350-21713950:+	ENSG00000078403	MLLT10	90.5	0.0	0.0	0.0	
15:40608847-40619011:+	ENSG00000137812	KNL1	90.5	0.0	0.0	20.1	
11:86001035-86031611:-	ENSG00000073921	PICALM	89.3	0.0	7.8	17.2	new
10:21681332-21682257:+	ENSG00000078403	MLLT10	0.0	89.0	34.2	0.0	
10:21713772-21727928:+	ENSG00000078403	MLLT10	87.8	69.9	15.5	60.1	
11:119232448-119232695:+	ENSG00000110395	CBL	87.8	0.0	7.6	0.0	
X:119633360-119637195:-	ENSG00000125354	SEPT6	87.8	0.0	0.0	0.0	
4:48643173-48643467:-	ENSG00000075539	FRYL	87.4	0.0	0.0	0.0	new
9:129899965-129900547:-	ENSG00000187239	FNBP1	0.0	87.2	37.4	0.0	
1:51363866-51366029:-	ENSG00000085832	EPS15	0.0	86.8	10.5	0.0	
12:26628033-26655852:-	ENSG00000123104	ITPR2	86.4	45.1	41.1	31.0	
11:85981745-86011136:-	ENSG00000073921	PICALM	0.0	7.2	9.5	84.7	
11:118497936-118499420:+	ENSG00000118058	KMT2A	84.4	8.8	16.4	0.0	

12:26550247-26561952:-	ENSG00000123104	ITPR2	84.4	6.0	5.5	15.0	
1:51440347-51448135:-	ENSG00000085832	EPS15	84.4	57.5	39.8	25.2	
15:85664563-85710645:+	ENSG00000170776	AKAP13	0.0	27.3	6.8	83.0	
11:86000643-86031611:-	ENSG00000073921	PICALM	0.0	9.0	12.2	82.5	new
9:129957360-129994958:-	ENSG00000187239	FNBP1	0.0	23.8	4.2	81.1	
6:17661653-17665385:-	ENSG00000124789	NUP153	0.0	0.0	80.8	0.0	
5:65528452-65529199:-	ENSG00000123219	CENPK	0.0	33.0	73.4	77.6	
11:85981745-85990399:-	ENSG00000073921	PICALM	0.0	26.5	37.4	76.1	
1:51403419-51408332:-	ENSG00000085832	EPS15	75.4	6.4	19.8	23.0	
6:89846562-89871388:+	ENSG00000118412	CASP8AP2	75.4	0.0	49.5	0.0	
18:50895809-50940386:+	ENSG00000082212	ME2	0.0	0.0	0.0	74.2	
11:120476661-120477526:+	ENSG00000196914	ARHGEF12	74.0	21.7	38.0	0.0	
5:142901935-142932125:+	ENSG00000145819	ARHGAP26	74.0	0.0	7.3	15.0	
6:17661653-17675770:-	ENSG00000124789	NUP153	74.0	25.8	22.1	0.0	
11:119271735-119274953:+	ENSG00000110395	CBL	0.0	73.9	44.4	22.8	
2:100104402-100105583:-	ENSG00000144218	AFF3	0.0	73.7	5.8	0.0	new
9:122984485-123020459:+	ENSG00000011454	RABGAP1	70.7	9.0	0.0	0.0	
X:119629318-119633492:-	ENSG00000125354	SEPT6	70.7	13.5	4.9	0.0	
9:122996041-123020459:+	ENSG00000011454	RABGAP1	0.0	6.3	4.2	70.4	
11:120473050-120477526:+	ENSG00000196914	ARHGEF12	0.0	40.3	69.8	0.0	
16:3736036-3745354:-	ENSG00000005339	CREBBP	0.0	25.5	18.2	68.5	
18:50918110-50925898:+	ENSG00000082212	ME2	0.0	20.9	5.1	68.1	
X:119629318-119629508:-	ENSG00000125354	SEPT6	0.0	68.0	57.6	0.0	
19:6230570-6270759:-	ENSG00000130382	MLLT1	0.0	21.6	67.6	60.1	
9:129895838-129908999:-	ENSG00000187239	FNBP1	0.0	9.0	67.6	0.0	
15:85664563-85684873:+	ENSG00000170776	AKAP13	67.2	51.6	15.3	58.5	
19:8554477-8555796:-	ENSG00000142347	MYO1F	0.0	66.9	53.5	39.5	
22:41135813-41140257:+	ENSG00000100393	EP300	0.0	65.1	30.8	30.4	
15:85682158-85693451:+	ENSG00000170776	AKAP13	0.0	64.9	0.0	45.6	
5:62347130-62355254:+	ENSG00000068796	KIF2A	0.0	64.6	31.7	26.7	
4:39837856-39845880:-	ENSG00000121892	PDS5A	0.0	8.5	19.5	64.5	
4:87084120-87091829:+	ENSG00000172493	AFF1	0.0	64.3	22.5	14.6	
1:51421786-51448135:-	ENSG00000085832	EPS15	0.0	23.4	37.4	63.5	
5:143014080-143057747:+	ENSG00000145819	ARHGAP26	0.0	0.0	22.0	61.9	
15:85664563-85682212:+	ENSG00000170776	AKAP13	61.6	6.6	0.0	0.0	
4:48590659-48609823:-	ENSG00000075539	FRYL	61.6	0.0	0.0	0.0	new
1:51394381-51421858:-	ENSG00000085832	EPS15	0.0	61.0	30.5	50.3	
22:41170406-41173784:+	ENSG00000100393	EP300	0.0	26.8	26.8	60.5	
6:108561086-108561829:+	ENSG00000118689	FOXO3	0.0	60.4	18.5	0.0	new
19:8548036-8553437:-	ENSG00000142347	MYO1F	0.0	0.0	0.0	60.3	new
4:39841948-39845880:-	ENSG00000121892	PDS5A	0.0	60.0	15.9	15.0	
5:132927121-132934941:-	ENSG00000072364	AFF4	0.0	40.4	58.3	36.8	
2:100006632-100011571:-	ENSG00000144218	AFF3	0.0	57.4	0.0	58.1	
4:39848851-39849652:-	ENSG00000121892	PDS5A	0.0	57.7	29.5	0.0	
22:40429606-40431480:-	ENSG00000196588	MKL1	0.0	57.0	0.0	0.0	
16:3749627-3770986:-	ENSG00000005339	CREBBP	0.0	12.8	56.9	29.0	

12:26415303-26443650:-	ENSG00000123104	ITPR2	0.0	14.5	0.0	56.3	
9:129924960-129929695:-	ENSG00000187239	FNBP1	55.0	0.0	10.3	0.0	new
5:142787460-142790458:+	ENSG00000145819	ARHGAP26	0.0	0.0	54.9	0.0	
5:142787460-142792773:+	ENSG00000145819	ARHGAP26	0.0	0.0	0.0	54.4	
19:8548036-8552164:-	ENSG00000142347	MYO1F	0.0	0.0	5.6	54.1	
18:50895809-50932360:+	ENSG00000082212	ME2	0.0	8.5	0.0	53.5	
2:203366877-203427269:+	ENSG00000138443	ABI2	0.0	30.0	52.6	27.2	
4:39872986-39890364:-	ENSG00000121892	PDS5A	0.0	16.0	52.4	48.5	
15:85710579-85741495:+	ENSG00000170776	AKAP13	0.0	0.0	52.2	0.0	new
19:8550160-8553437:-	ENSG00000142347	MYO1F	0.0	0.0	26.6	51.9	new
3:188609161-188609844:+	ENSG00000145012	LPP	0.0	9.0	45.4	51.7	
2:241342992-241346249:+	ENSG00000168385	SEPT2	0.0	51.7	44.8	25.5	
5:65528452-65551636:-	ENSG00000123219	CENPK	0.0	14.3	13.8	51.2	
11:74118228-74123135:-	ENSG00000168014	C2CD3	0.0	49.2	51.2	0.0	
4:39837478-39842056:-	ENSG00000121892	PDS5A	0.0	15.0	50.6	26.8	
15:85655417-85664755:+	ENSG00000170776	AKAP13	0.0	0.0	0.0	49.5	
11:86014870-86031611:-	ENSG00000073921	PICALM	0.0	0.0	0.0	49.0	new
11:120406118-120407823:+	ENSG00000196914	ARHGEF12	0.0	19.5	48.9	0.0	
15:85710579-85719326:+	ENSG00000170776	AKAP13	0.0	48.5	34.5	0.0	
5:62363178-62363899:+	ENSG00000068796	KIF2A	0.0	0.0	48.0	0.0	
1:51459041-51465326:-	ENSG00000085832	EPS15	0.0	47.9	7.6	0.0	
17:18285142-18285520:-	ENSG00000177302	TOP3A	0.0	36.1	22.0	47.5	
18:50924098-50932360:+	ENSG00000082212	ME2	0.0	23.9	46.6	0.0	
15:85664563-85693451:+	ENSG00000170776	AKAP13	0.0	46.3	30.6	18.9	
2:241342992-241348191:+	ENSG00000168385	SEPT2	0.0	46.2	39.7	44.3	
11:85996826-86026367:-	ENSG00000073921	PICALM	0.0	0.0	44.7	30.4	new
3:188341663-188524787:+	ENSG00000145012	LPP	0.0	0.0	44.5	32.3	
5:62347130-62350120:+	ENSG00000068796	KIF2A	0.0	43.3	43.5	0.0	
18:50895809-50912950:+	ENSG00000082212	ME2	0.0	43.1	12.6	16.7	
18:50895809-50925898:+	ENSG00000082212	ME2	0.0	6.4	7.3	43.1	
5:142894238-142932125:+	ENSG00000145819	ARHGAP26	0.0	0.0	42.8	0.0	new
1:51394381-51409696:-	ENSG00000085832	EPS15	0.0	0.0	9.1	42.2	
1:51402435-51406108:-	ENSG00000085832	EPS15	0.0	0.0	42.1	17.7	
6:17669293-17675033:-	ENSG00000124789	NUP153	0.0	42.0	14.8	17.3	
15:85664563-85669830:+	ENSG00000170776	AKAP13	0.0	41.6	4.9	16.7	
5:62363178-62366481:+	ENSG00000068796	KIF2A	0.0	0.0	41.3	0.0	
4:48556978-48557712:-	ENSG00000075539	FRYL	0.0	6.4	8.5	41.2	
3:188325781-188335197:+	ENSG00000145012	LPP	0.0	0.0	0.0	40.5	
15:85655417-85655787:+	ENSG00000170776	AKAP13	0.0	34.3	40.3	0.0	
5:62362450-62363899:+	ENSG00000068796	KIF2A	0.0	18.5	40.3	18.2	
11:85996826-86007583:-	ENSG00000073921	PICALM	0.0	0.0	24.0	40.1	
18:50895809-50924212:+	ENSG00000082212	ME2	0.0	0.0	0.0	38.9	new
15:57231152-57263274:+	ENSG00000140262	TCF12	0.0	0.0	38.1	0.0	
15:85682158-85708086:+	ENSG00000170776	AKAP13	0.0	0.0	0.0	38.0	
11:120406118-120409450:+	ENSG00000196914	ARHGEF12	0.0	37.9	0.0	0.0	
16:3749627-3758972:-	ENSG00000005339	CREBBP	0.0	0.0	0.0	37.9	

1:51402435-51447105:-	ENSG00000085832	EPS15	0.0	0.0	4.2	37.9	new
15:85662388-85684873:+	ENSG00000170776	AKAP13	0.0	0.0	0.0	37.6	new
3:45703438-45719601:+	ENSG00000211456	SACM1L	0.0	0.0	0.0	37.6	
4:48539971-48542121:-	ENSG00000075539	FRYL	0.0	0.0	0.0	37.6	new
12:26769349-26790227:-	ENSG00000123104	ITPR2	0.0	29.8	37.3	0.0	
22:41140140-41141222:+	ENSG00000100393	EP300	0.0	37.3	0.0	16.7	
18:50895809-50908196:+	ENSG00000082212	ME2	0.0	37.0	6.8	17.3	
22:41154995-41157408:+	ENSG00000100393	EP300	0.0	37.0	32.7	0.0	
4:39902347-39925933:-	ENSG00000121892	PDS5A	0.0	0.0	0.0	36.3	new
4:39913611-39922748:-	ENSG00000121892	PDS5A	0.0	36.2	13.1	19.0	
11:96091892-96119106:-	ENSG00000184384	MAML2	0.0	0.0	0.0	36.2	new
5:142932047-143057747:+	ENSG00000145819	ARHGAP26	0.0	0.0	0.0	36.0	
4:76996425-77030970:+	ENSG00000138758	SEPT11	0.0	0.0	4.2	35.8	
17:9969677-10019897:-	ENSG00000007237	GAS7	0.0	0.0	0.0	35.7	
1:51363866-51447105:-	ENSG00000085832	EPS15	0.0	0.0	35.4	0.0	new
2:241335126-241343093:+	ENSG00000168385	SEPT2	0.0	0.0	35.3	0.0	
6:89854817-89855544:+	ENSG00000118412	CASP8AP2	0.0	9.5	35.2	0.0	
22:41131388-41141222:+	ENSG00000100393	EP300	0.0	0.0	34.9	0.0	
16:3740399-3751806:-	ENSG00000005339	CREBBP	0.0	21.3	21.9	34.6	
7:74338448-74339004:+	ENSG00000106665	CLIP2	0.0	34.6	0.0	0.0	
12:26621123-26632059:-	ENSG00000123104	ITPR2	0.0	18.5	27.9	34.5	
22:41125864-41131633:+	ENSG00000100393	EP300	0.0	13.5	11.4	34.5	
5:142780269-142792773:+	ENSG00000145819	ARHGAP26	0.0	0.0	0.0	34.3	
11:85971476-85983973:-	ENSG00000073921	PICALM	0.0	0.0	34.3	0.0	
10:21670449-21681376:+	ENSG00000078403	MLLT10	0.0	0.0	0.0	34.3	new
5:132927121-132937193:-	ENSG00000072364	AFF4	0.0	5.7	0.0	34.1	
X:119640692-119663677:-	ENSG00000125354	SEPT6	0.0	26.8	33.9	0.0	
15:85662388-85710645:+	ENSG00000170776	AKAP13	0.0	10.2	8.1	33.6	new
9:122989297-122990213:+	ENSG00000011454	RABGAP1	0.0	10.4	33.6	0.0	
3:45735235-45739644:+	ENSG00000211456	SACM1L	0.0	0.0	0.0	33.3	new
2:232747615-232761436:+	ENSG00000204120	GIGYF2	0.0	33.2	32.9	17.4	
19:8553139-8553437:-	ENSG00000142347	MYO1F	0.0	32.8	32.5	21.2	
18:50916168-50932360:+	ENSG00000082212	ME2	0.0	0.0	0.0	32.6	
1:51405905-51421858:-	ENSG00000085832	EPS15	0.0	0.0	32.6	0.0	new
2:99837477-100008932:-	ENSG00000144218	AFF3	0.0	30.1	0.0	32.2	new
11:992503-994245:+	ENSG00000183020	AP2A2	0.0	26.1	32.0	15.0	
5:143014080-143121147:+	ENSG00000145819	ARHGAP26	0.0	0.0	0.0	31.8	new
11:96132738-96257116:-	ENSG00000184384	MAML2	0.0	0.0	0.0	31.7	new
4:87108159-87115299:+	ENSG00000172493	AFF1	0.0	15.7	0.0	31.7	
11:120475340-120477526:+	ENSG00000196914	ARHGEF12	0.0	31.6	5.1	0.0	
4:39837478-39838208:-	ENSG00000121892	PDS5A	0.0	0.0	31.6	0.0	
3:155919233-155925366:+	ENSG00000163655	GMPS	0.0	31.5	13.1	0.0	
10:26764510-26765318:-	ENSG00000136754	ABI1	0.0	31.4	13.7	0.0	
9:20413721-20415550:-	ENSG00000171843	MLLT3	0.0	0.0	31.2	0.0	new
11:74074253-74109152:-	ENSG00000168014	C2CD3	0.0	0.0	31.2	0.0	new
9:129902869-129908999:-	ENSG00000187239	FNBP1	0.0	31.0	7.8	0.0	

11:85981745-86007583:-	ENSG00000073921	PICALM	0.0	0.0	0.0	30.8	
15:40645656-40647074:+	ENSG00000137812	KNL1	0.0	0.0	0.0	30.7	new
11:85990250-86003451:-	ENSG00000073921	PICALM	0.0	0.0	15.7	30.6	
19:8536252-8553437:-	ENSG00000142347	MYO1F	0.0	0.0	4.2	30.4	
22:41125864-41173784:+	ENSG00000100393	EP300	0.0	0.0	0.0	30.4	
3:155893518-155925366:+	ENSG00000163655	GMPS	0.0	0.0	0.0	30.4	
2:241324216-241335212:+	ENSG00000168385	SEPT2	0.0	30.1	0.0	0.0	
12:26595465-26655852:-	ENSG00000123104	ITPR2	0.0	9.5	0.0	30.0	new
18:50916168-50925898:+	ENSG00000082212	ME2	0.0	0.0	0.0	30.0	new
15:85710579-85718159:+	ENSG00000170776	AKAP13	0.0	0.0	29.9	0.0	
7:5370375-5371364:-	ENSG00000182095	TNRC18	0.0	15.2	29.6	27.5	
2:232811244-232812491:+	ENSG00000204120	GIGYF2	0.0	29.4	5.4	0.0	
6:17665239-17669028:-	ENSG00000124789	NUP153	0.0	7.2	29.4	29.0	
9:20413721-20456786:-	ENSG00000171843	MLLT3	0.0	5.8	29.2	0.0	
3:155893518-155906263:+	ENSG00000163655	GMPS	0.0	0.0	28.9	28.6	
15:85734992-85736134:+	ENSG00000170776	AKAP13	0.0	28.9	4.2	0.0	
2:99649626-100011571:-	ENSG00000144218	AFF3	0.0	0.0	0.0	28.6	new
5:62347130-62366481:+	ENSG00000068796	KIF2A	0.0	0.0	28.6	0.0	
11:116759074-116765446:-	ENSG00000137656	BUD13	0.0	0.0	0.0	28.4	
11:85978070-85981228:-	ENSG00000073921	PICALM	0.0	0.0	0.0	28.4	
22:41170406-41176528:+	ENSG00000100393	EP300	0.0	0.0	0.0	28.4	
7:5345562-5394595:-	ENSG00000182095	TNRC18	0.0	0.0	0.0	28.4	new
9:122996041-123010528:+	ENSG00000011454	RABGAP1	0.0	20.2	24.1	28.4	
3:188755058-188760282:+	ENSG00000145012	LPP	0.0	0.0	28.1	17.4	
4:87114367-87115299:+	ENSG00000172493	AFF1	0.0	0.0	27.7	19.0	
12:26631866-26655852:-	ENSG00000123104	ITPR2	0.0	27.7	0.0	0.0	
9:20448123-20456786:-	ENSG00000171843	MLLT3	0.0	0.0	27.5	0.0	
22:41141048-41157408:+	ENSG00000100393	EP300	0.0	0.0	0.0	27.3	
11:118477967-118482495:+	ENSG00000118058	KMT2A	0.0	27.2	10.6	0.0	
11:118494288-118499420:+	ENSG00000118058	KMT2A	0.0	11.5	0.0	27.1	
4:39904040-39925933:-	ENSG00000121892	PDS5A	0.0	0.0	24.8	26.9	
5:65521475-65529199:-	ENSG00000123219	CENPK	0.0	26.7	7.9	0.0	
19:8551740-8555796:-	ENSG00000142347	MYO1F	0.0	0.0	16.0	26.7	new
4:48593930-48609067:-	ENSG00000075539	FRYL	0.0	0.0	26.7	0.0	
15:40644997-40652105:+	ENSG00000137812	KNL1	0.0	0.0	0.0	26.6	
10:68644698-68652594:+	ENSG00000138336	TET1	0.0	0.0	26.6	0.0	
11:85981129-85983973:-	ENSG00000073921	PICALM	0.0	26.6	14.5	22.2	
10:21681332-21727928:+	ENSG00000078403	MLLT10	0.0	0.0	26.5	0.0	
22:41149761-41152350:+	ENSG00000100393	EP300	0.0	0.0	26.5	0.0	new
1:51440347-51447105:-	ENSG00000085832	EPS15	0.0	0.0	26.4	0.0	
15:57091789-57095163:+	ENSG00000140262	TCF12	0.0	0.0	26.3	0.0	
4:87084120-87094969:+	ENSG00000172493	AFF1	0.0	26.2	19.7	0.0	
22:41135813-41141222:+	ENSG00000100393	EP300	0.0	0.0	25.9	0.0	
19:8541906-8548317:-	ENSG00000142347	MYO1F	0.0	0.0	0.0	25.9	new
3:188484592-188609844:+	ENSG00000145012	LPP	0.0	0.0	0.0	25.9	
6:17624561-17649300:-	ENSG00000124789	NUP153	0.0	21.9	8.9	25.9	

16:3791981-3793626:-	ENSG00000005339	CREBBP	0.0	25.3	10.3	0.0	
9:129895838-129903001:-	ENSG00000187239	FNBP1	0.0	0.0	25.2	0.0	
11:119275997-119287946:+	ENSG00000110395	CBL	0.0	7.2	0.0	25.2	
4:39917048-39925933:-	ENSG00000121892	PDS5A	0.0	19.5	0.0	25.2	
5:132883340-132889173:-	ENSG00000072364	AFF4	0.0	13.4	0.0	25.2	
5:62357691-62361529:+	ENSG00000068796	KIF2A	0.0	0.0	25.1	0.0	
12:26681874-26695650:-	ENSG00000123104	ITPR2	0.0	5.9	25.0	0.0	new
2:203366877-203402734:+	ENSG00000138443	ABI2	0.0	0.0	24.9	16.7	
17:9940126-9959255:-	ENSG00000007237	GAS7	0.0	0.0	13.3	24.5	
11:120406118-120431911:+	ENSG00000196914	ARHGEF12	0.0	0.0	24.3	0.0	
9:122998597-123020459:+	ENSG00000011454	RABGAP1	0.0	0.0	24.3	0.0	
15:85662388-85708086:+	ENSG00000170776	AKAP13	0.0	0.0	24.2	0.0	new
11:116762553-116765446:-	ENSG00000137656	BUD13	0.0	24.2	0.0	16.5	
15:85684741-85710645:+	ENSG00000170776	AKAP13	0.0	24.0	8.4	0.0	

Supplementary Table 3. Metadata for the three MLLre samples. For each sample, the translocation is specified indicating the TPG and the exons flanking the breakpoint; key information on the RNA-seq data is also provided (linearly unmapped reads were used for circRNA quantification).

Sample	Leukemia	Sex	Age at DX	Fusion	RNA-seq data			
					Library	Sequencer	Depth	Linearly mapped and unmapped reads
THP1 cells (SRR3239 808)	AML	M	1	KMT2A/MLL ex8-ex6 AF9/MLLT3	rRNA depletion	NextSeq500	50 M, single end, 75 nt reads; 83% passed quality control	28 M<br>14 M
x039	c-ALL	F	0	KMT2A/MLL ex9-ex2 ENL/MLLT1	rRNA depletion	HiSeq2000	118 M, paired end, 100 nt reads; 64% passed quality control	72 M<br>4 M
x142	Pro B-ALL	M	0.9	KMT2A/MLL ex8-ex4 ENL/MLLT1	rRNA depletion	HiSeq2000	144 M, paired end, 100 nt reads; 60% passed quality control	85 M<br>2 M

Supplementary Figure 1. Expression level in the considered four blood cell populations of the 16,606 highly expressed circRNAs, separating circRNAs expressed from *MLL* recombinome (327 circRNAs from 54 genes) and from the other genes (16,005 circRNAs from 5,116 genes); 274 circRNAs expressed from intergenic regions were not included in the analysis.

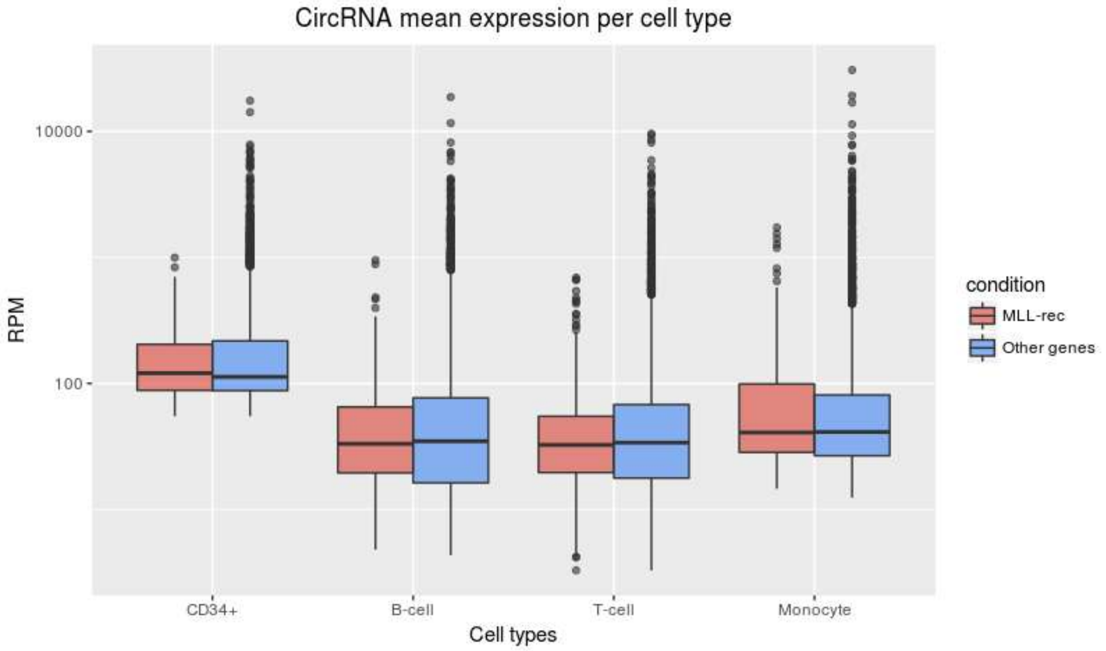

Supplementary Figure 2. Average expression in the considered blood cell populations of the 33 circRNAs most highly expressed (10% top ranking), which derived from 21 distinct genes of the *MLL* recombinome. CircRNAs are ordered by the highest mean expression among cell types. S, stem cells; B, B-cells; T, T-cells; M, monocytes.

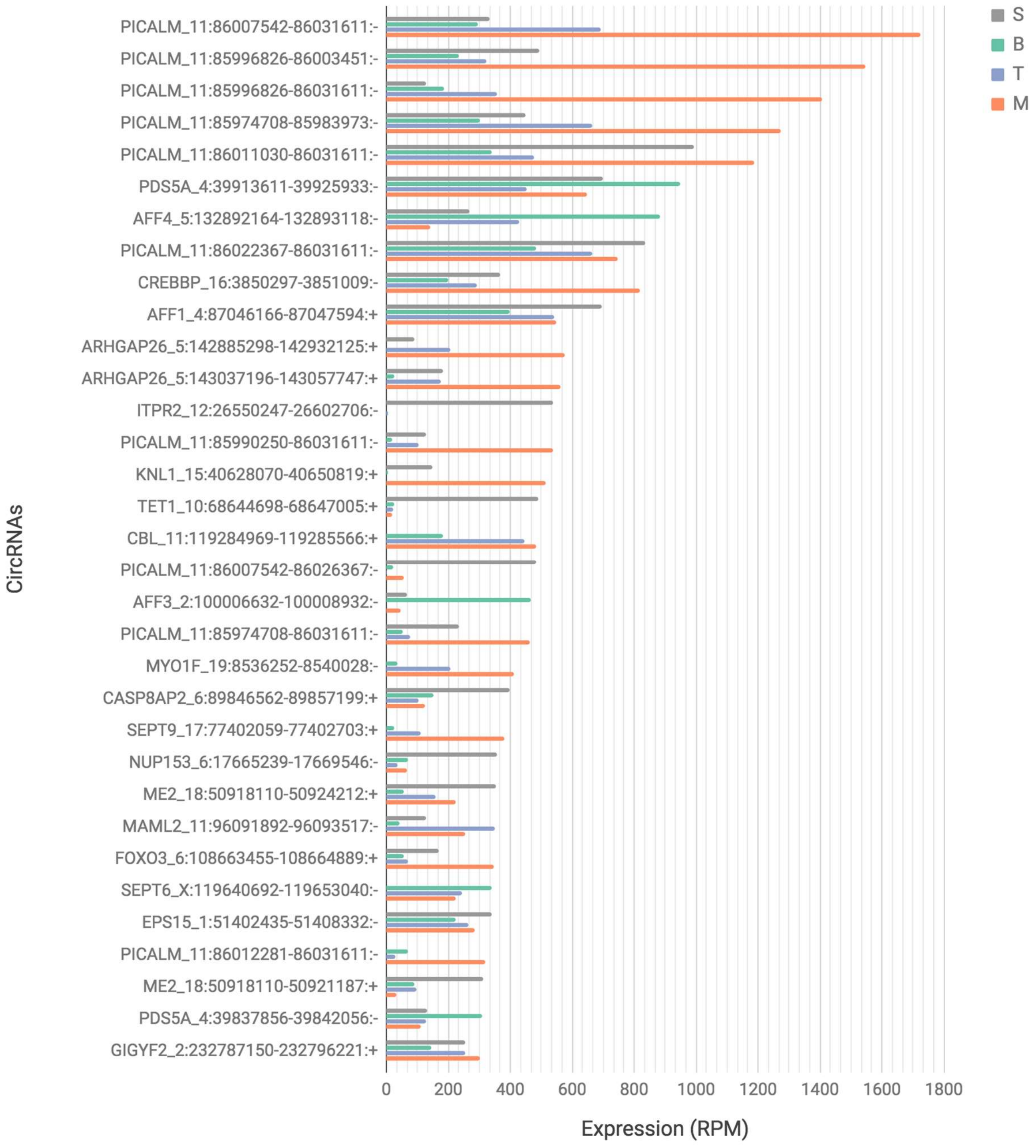

Supplementary Figure 3. Number of circular isoforms of the 54 genes of the *MLL* recombinome, and of the 5,116 other genes with circRNAs highly expressed in blood cells, A) considering all the observed values, and B) discretizing the number of circRNAs per gene in five classes, as shown in the legend.

A

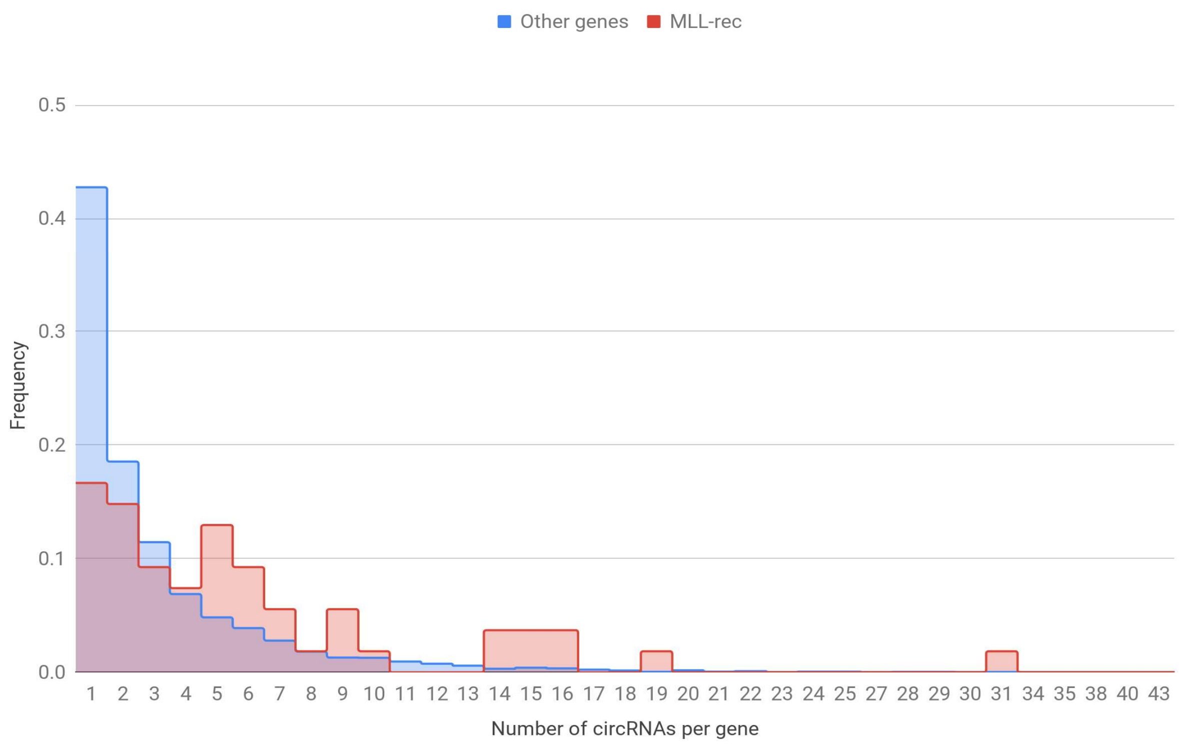

B

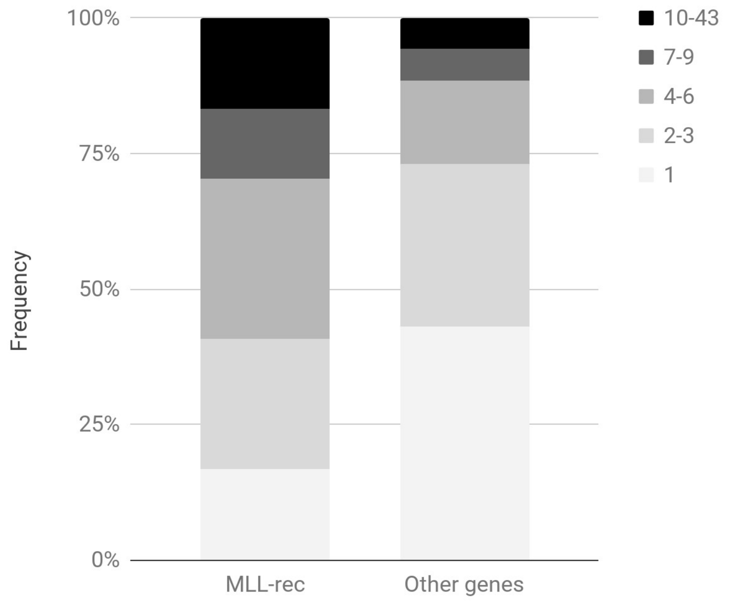

Supplementary Figure 4. Cell type-specificity of circRNAs. A) CircRNAs expressed by genes of the MLLrec in the four considered blood cell populations; B) Genes expressing circRNAs in different blood cell populations (a gene was classified according to the expression of all the circular isoforms of the gene, e.g. only one gene had one circRNA detected only in stem cells, whereas two genes had circRNAs expressed in stem and both B- and T-cells). C) Expression profile of the 16 alternative circEPS15.

A

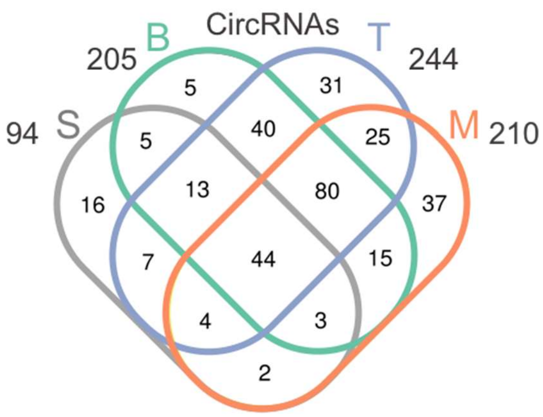

B

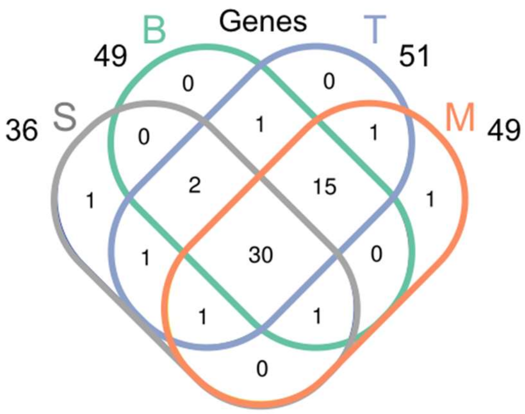

C

CircEPS15 isoforms	Expression (RPM) S	B	T	M
circEPS15_1:51402435-51408332:-	338	223	266	285
circEPS15_1:51394381-51448135:-	62	25	31	242
circEPS15_1:51405905-51408332:-		122	108	36
circEPS15_1:51461091-51465326:-		96	67	
circEPS15_1:51363866-51366029:-		87	10	
circEPS15_1:51440347-51448135:-	84	57	40	25
circEPS15_1:51403419-51408332:-	75	6	20	23
circEPS15_1:51421786-51448135:-		23	37	63
circEPS15_1:51394381-51421858:-		61	31	50
circEPS15_1:51459041-51465326:-		48	8	
circEPS15_1:51394381-51409696:-			9	42
circEPS15_1:51402435-51406108:-			42	18
circEPS15_1:51402435-51447105:-			4	38
circEPS15_1:51363866-51447105:-			35	
circEPS15_1:51405905-51421858:-			33	
circEPS15_1:51440347-51447105:-			26	
